# Precision Medicine in Fatty Liver Disease/Non-Alcoholic Fatty Liver Disease

**DOI:** 10.3390/jpm13050830

**Published:** 2023-05-14

**Authors:** Laura Valenzuela-Vallejo, Despina Sanoudou, Christos S. Mantzoros

**Affiliations:** 1 Department of Medicine, Beth-Israel Deaconess Medical Center, Harvard Medical School, Boston, MA 02215, USA; lvalenz1@bidmc.harvard.edu; 2 Clinical Genomics and Pharmacogenomics Unit, 4(th) Department of Internal Medicine, Attikon Hospital, Medical School, National and Kapodistrian University of Athens, 11527 Athens, Greece; dsanoudou@med.uoa.gr; 3 Center for New Biotechnologies and Precision Medicine, Medical School, National and Kapodistrian University of Athens, 11527 Athens, Greece; 4 Molecular Biology Division, Biomedical Research Foundation of the Academy of Athens, 11527 Athens, Greece; 5 Department of Medicine, Boston VA Healthcare System, Boston, MA 02130, USA

**Keywords:** precision medicine, non-alcoholic fatty liver disease (NAFLD), non-alcoholic steatohepatitis (NASH), fatty liver disease (FLD), metabolic-associated fatty liver disease (MAFLD), genetics, metabolism

## Abstract

Non-alcoholic fatty liver disease (NAFLD) is the most prevalent chronic liver disease, and is related to fatal and non-fatal liver, metabolic, and cardiovascular complications. Its non-invasive diagnosis and effective treatment remain an unmet clinical need. NAFLD is a heterogeneous disease that is most commonly present in the context of metabolic syndrome and obesity, but not uncommonly, may also be present without metabolic abnormalities and in subjects with normal body mass index. Therefore, a more specific pathophysiology-based subcategorization of fatty liver disease (FLD) is needed to better understand, diagnose, and treat patients with FLD. A precision medicine approach for FLD is expected to improve patient care, decrease long-term disease outcomes, and develop better-targeted, more effective treatments. We present herein a precision medicine approach for FLD based on our recently proposed subcategorization, which includes the metabolic-associated FLD (MAFLD) (i.e., obesity-associated FLD (OAFLD), sarcopenia-associated FLD (SAFLD, and lipodystrophy-associated FLD (LAFLD)), genetics-associated FLD (GAFLD), FLD of multiple/unknown causes (XAFLD), and combined causes of FLD (CAFLD) as well as advanced stage fibrotic FLD (FAFLD) and end-stage FLD (ESFLD) subcategories. These and other related advances, as a whole, are expected to enable not only improved patient care, quality of life, and long-term disease outcomes, but also a considerable reduction in healthcare system costs associated with FLD, along with more options for better-targeted, more effective treatments in the near future.

## 1. Introduction

Non-alcoholic fatty liver disease (NAFLD) is characterized by excess fat deposition in the liver (>5% based on histology or 5.6% based on MRI), and its diagnosis is based on the exclusion of other causes of liver abnormalities, particularly excessive alcohol use [1]. The presence of steatosis characterizes NAFLD’s early stage, known as NAFL. In 20% of this population, NAFL can develop into non-alcoholic steatohepatitis (NASH), which includes inflammation that leads to hepatocyte injury (ballooning). In turn, 20% of the subjects with NASH can develop liver complications such as fibrosis, cirrhosis, and liver failure, as well as atherosclerotic cardiovascular disease (ASCVD), its primary cause of mortality [2,3].

NAFLD is becoming the most prevalent chronic liver disease affecting more than 25% of the population. The rising burden of NAFLD is intimately linked to the global increase in obesity and cardiometabolic abnormalities [4]. NAFLD’s leading risk factors include obesity, which is present in 51.34% of NAFLD patients, insulin resistance and type 2 diabetes mellitus (T2DM) in 22.51%, dyslipidemia in 22.51%, and hypertension in 39.34% [5,6]. In essence, the association between NAFLD, metabolic derangements, and ASCVD have led to the use of the term metabolic-associated fatty liver disease (MAFLD), suggesting that NAFLD could be the hepatic manifestation of the metabolic syndrome [7,8].

However, NAFLD is a complex condition that cannot be viewed as simply the consequence of metabolic syndrome. For example, non-obese people or subjects without typical cardiometabolic risk factors can develop NAFLD. Meanwhile, other established risk factors for this condition include sarcopenia, lipodystrophy, genetic abnormalities, genetic background/at-risk ethnicities, and the use of some pharmacological agents, among others [3,9,10]. Due to this broad spectrum of underlying factors leading to NAFLD, recently, a more precise pathophysiology-based classification of NAFLD has been proposed: the replacement of a negative term of what the disease is not (non-alcoholic) with a physiology-based “umbrella” definition of fatty liver disease (FLD), which in turn is more precisely subclassified on the basis of specific etiologies. More specifically, this novel proposed classification includes metabolic-associated FLD (MAFLD), which is divided into obesity-associated FLD (OAFLD), sarcopenia-associated FLD (SAFLD, and lipodystrophy-associated FLD (LAFLD). It also proposes a genetics-associated FLD (GAFLD), multiple or unknown causes of FLD (XAFLD), alcohol-associated fatty liver disease (AAFLD), as well as a more advanced stage fibrotic FLD (FAFLD), and end-stage FLD (ESFLD) subcategories [3]. In addition, some patients could present combined causes of FLD (CAFLD) that need to be identified and included in the clinical approach, e.g., have both AAFLD due to excess alcohol intake, and central obesity (O-MAFLD) and/or S-MAFLD due to sarcopenia [3].

The novel pathophysiology-based classification proposed for FLD offers a precision medicine (PM) approach toward an improved diagnosis, a more accurate prognosis, and more effective treatments. More specifically, through the combinatorial consideration of all the different levels of medically relevant information, including epidemiological, clinical, and laboratory information, the distinct disease causes and patient needs can be pinpointed, opening the pathway for the design of personalized and, therefore, more effective therapeutic strategies. This review focuses on the current and promising future role of PM in the classification, diagnosis, and treatment of FLD.

### Delineating the Distinct FLD Pathophysiological Entities

The pathophysiology of NAFLD is heterogeneous and is influenced by multiple factors, including lifestyle, genetics, epigenetics, and metabolic dysfunction. These factors may be present in different combinations and with variable impacts on each individual. However, the unifying underlying basis is that all the primary processes lead to an excessive accumulation of fatty acids (FA) in the liver, which drives hepatocyte damage and may lead to liver fibrosis and cirrhosis [11]. More specifically, hepatocytes are saturated with FA, which may either be transported to the liver from the adipose tissue (AT) after lipolysis or are produced de novo inside the hepatocytes; both sources are altered in the presence of genetic abnormalities, insulin resistance, or an unhealthy lifestyle [12]. The FA stored in the liver can be processed through several different pathways, including b-oxidation inside the mitochondria or peroxisomes, oxidation by the cytochrome P-450 enzymes in the smooth endoplasmic reticulum, or transformation into triglycerides (TG) that are either released as very low-density lipoproteins (VLDL) or stored in hepatocytes. The excessive FA accumulation leads to oxidative and endoplasmic reticulum stress, inflammation, inflammasome activation, and ultimately hepatocyte fibrosis and apoptosis [13]. 

Even though liver fat accumulation is the shared denominator across all NAFLD patients, the underlying molecular setting, initial pathogenetic steps, risk factors, or triggers may vary considerably across individuals. Hence, it has been proposed that the NAFLD nomenclature should be revised based on its pathophysiology and be made similar to other conditions where a broader term encompasses multiple significantly different disease entities, such as diabetes mellitus (e.g., Type 1, Type 2, MODY) or hyperthyroidism (e.g., Graves’ disease, extra- or intrathyroidal causes, adenomas, or multinodular goiter) [3]. A more accurate classification as the novel FLD pathophysiology-based grouping can pave the way for broader utilization of more appropriate screening tools, enable the early identification of individuals at risk, and facilitate the timely use of individualized and, therefore, more appropriate preventive strategies. Furthermore, it will open the way for the development—and facilitate the implementation—of targeted diagnostic and therapeutic strategies, ultimately offering an improved quality of life and a significantly reduced disease burden. These combined approaches serve as the core of PM.

## 2. Precision Medicine in FLD

### 2.1. Genetics-Associated FLD Disease (GAFLD) and Precision Medicine

#### 2.1.1. GAFLD Pathophysiology

GAFLD is proposed to be characterized by the absence of primary dysmetabolic abnormalities and obesity and is thus considered to be driven mainly by genetic variants contributing to liver fat accumulation and damage [14,15,16,17]. A multitude of genes and genetic variants are being investigated for their impact on predisposition to NAFLD development, with prime examples described below [18,19,20]. 

Patatin-like phospholipase domain-containing 3 (PNPLA3), also known as adiponutrin (ADPN), is one of the most studied genes in relationship to hepatic steatosis and NAFLD progression. In vitro and animal studies have proposed that when the PNPLA3 common nonsynonymous variant (rs738409 C > G; p.I148M) is present, there is an increased lipid droplet accumulation in hepatocytes, lipolysis impairment (by decreased hydrolase activity), as well as increase in pro-inflammatory and fibrosis processes [19,21,22]. In addition, the association of this variant with hepatic steatosis has been observed through extensive GWAS studies of various ethnicities, which also unveiled inter-ethnic variability to NAFLD susceptibility [20]. 

Additionally, in the glucokinase regulator (GCKR) gene, the rs1260326 common missense loss-of-function variant (p.P446L) has been associated with a decrease in the glucokinase in response to fructose-6-phosphate, an increased hepatic glucose uptake, and malonyl-CoA production. Through these effects, the rs1260326 variant is believed to drive the continuous activation of de novo lipogenesis-related enzymes and alter TG secretion, ultimately leading to hepatic lipid accumulation [20,23,24,25]. Furthermore, in the transmembrane 6 superfamily member 2 (TM6SF2) gene, which controls cholesterol synthesis in hepatocytes and the secretion of lipoproteins, the rs58542926 variant (p.E167K) has been associated with altered mechanisms that drive an increase in liver fat content, fibrosis, and HCC [20]. 

In the microsomal TG transfer protein (MTTP), variants such as the rs2306986 (c.294G > C, p.E98D) and rs1800591 (−493G > T) can lead to increased hepatic TG content and impaired production/transportation of very low-density lipoprotein (VLDL), and thus a higher susceptibility to NAFLD [18,19,26,27]. Moreover, the rs641738 (C > T) variant of the membrane-bound O-acyltransferase domain-containing protein 7 (MBOAT7), which is highly expressed in liver and inflammatory cells, has also been associated with hepatic fat content, a higher risk of inflammation, more severe liver damage, increased risk of fibrosis, and HCC [20]. It has been proposed that these effects may be mediated by liver phosphatidylinositol acyl-chain remodeling [28]. Similarly, multiple single nucleotide polymorphisms (SNPs) in the 11β-hydroxysteroid-dehydrogenase 1 (11β-HSD1) gene, such as the rs2235543, rs12565406, and rs4844880, have been strongly correlated to NAFLD pathogenesis and liver fat content (*p* = 0.0002, *p* = 0.001, and *p* = 0.0009, respectively) [29]. 

On the contrary, loss-of-function variants on the hydroxysteroid (17-beta) dehydrogenase-13 (HSD17B13) gene, such as the rs72613567, have been proposed to mitigate NAFLD’s progression to NASH, fibrosis, and hepatocellular carcinoma (HCC) by the loss of its enzymatic activity, which leads to increased retinol–binding protein (RBP4)–transthyretin (TTR) transport from hepatocytes [18,30,31,32]. Other variants associated with decreased lipid and transaminase levels include the A286V missense variant of the peroxisome proliferator-activated (PPAR) alpha receptor, which is highly expressed in the liver as part of the nuclear receptors’ superfamily and is a crucial regulator of lipid metabolism, beta-oxidation, and hepatic FA transport, as well as the G171A variant of the nuclear receptor subfamily 0 group B member 2 (SHP) [18]. All these variants need to be further studied to better determine their precise role in NAFLD pathophysiology in humans [33]. 

#### 2.1.2. Genetic Testing

The genetic basis of GAFLD is not fully delineated, and thus, clinical genetic testing is not currently available. However, an example of the attempts toward presymptomatic genetic testing for FLD is a genetic risk score (GRS) encompassing 47 SNPs associated with NAFLD for examining FLD risk in multiple ethnic groups. Statistically significant associations with NAFLD risk were found (but varied by populations) in 11 SNPs, including the rs738409 (*PNPLA3*), rs58542926 (*TM6SF2*), rs1260326 (*GCKR*), rs13118664 (*HSD17B13*), rs4808199 (*GATAD2A*), rs2954021 (*TRIB2*), rs4240624 (*PPP1R3B*), rs10883437 (*CPN1*), rs10883451 (*ERLIN1*), rs429358 (apolipoprotein E (*APOE*)), and rs641738 (*MBOAT7*)). The authors found that as the GRS increased, the overall NAFLD risk was higher, and this observation had the largest magnitude of association in Latinos. Of note, some populations have a higher risk of NAFLD development, and the disease burden varies across different geographical locations [34,35]. For instance, the Geography of Genetic Variants (GGV) browser shows a higher frequency of risk-G-rs738409 alleles and a low frequency of the G-rs6834314 protective alleles in several populations [36]. Thus, genetic biomarkers can hold considerable promise toward more accurate disease detection and prognosis in different populations [19]. 

#### 2.1.3. Therapeutic Strategies

The delineation of the genetic basis for GAFLD also opens new therapeutic avenues. Strategies include biological therapies for gene silencing, which can be performed by degrading targeted mRNAs using interfering RNA (RNAi) and antisense oligonucleotides (ASOs) [37]. The ASOs can skip exons and mediate the removal of mutant or deleterious exon sequences in the pre-mRNA, thus leading to protein function rescue [38]. Some representative examples of novel targeted therapeutic approaches that have reached clinical trials include RNAi methods to silence the previously mentioned HSD17B13 gene. They have been launched into phase I/II clinical trials for NAFLD, significantly downregulating the liver expression of HSD17B13 mRNA and protein, leading to marked reduction in transaminase levels (clinical trials: NCT04565717; NCT04202354) [30]. 

More specifically, studies with the ARO-HSD, an RNAi therapeutic designed to reduce the expression of HSD17B13 mRNA in hepatocytes, have shown good tolerance in humans and have led to significant mean alanine aminotransferase changes at 71 days of treatment: −7.7% (25 mg), −39.3% (100 mg), and −42.3% (200 mg) (*p* < 0.001 for pooled cohorts) [39]. The randomized, placebo-controlled, double-blinded, multicenter study with the AZD2693 molecule that aims to lower the mRNA expression of the PNPLA3 148M variant is currently being conducted and is in the recruiting phase of homozygote subjects with the variant (ClinicalTrials.gov Identifier: NCT04483947). This trial is still in the early phases, and long-term follow-up is required to determine its effects on FLD [30].

In preclinical studies, knock-in mouse models with the previously mentioned human PNPLA3 i148M gene variant resulted in decreased liver steatosis (*p* = 0.038) in the liver inflammation score (*p* = 0.018) and fibrosis stages (*p* = 0.031) in homozygous PNPLA3 i148M/M knock-in mice. However, in mice fed with a NASH-inducing diet, it reduced the liver steatosis index (LSI) and the NAFLD activity score (NAS) independently of the PNPLA3 genotype [40]. Other promising targets are being pursued in animal models and could also offer a potential future option for FLD treatment. However, several challenges are still faced in developing these biological therapies before they can transition to clinical practice [32,41,42,43,44].

Overall, current evidence points to GAFLD as a distinct entity under FLD. Significant progress has been made toward delineating its etiology, with multiple genetic variants showing a strong association with the disease development. Although a fine delineation of this subcategory of FLD combined with the ongoing mapping of its pathogenetic mechanisms is eagerly awaited, it is promising to enable significant improvements at the predictive, diagnostic, prognostic, and ultimately personalized treatment levels. 

### 2.2. Metabolic-Associated Fatty Liver Disease (MAFLD) and Its Subcategories in Precision Medicine

As mentioned, MAFLD occurs in the context of robust metabolic dysregulation or unhealthy lifestyles. Patients with MAFLD can present a variety of different phenotypes, such as: obesity and metabolic syndrome; lipodystrophy and profound dysfunctional fat; normal weight, yet with insulin resistance or decreased muscle mass; and other endocrinopathies [45]. As a consequence, MAFLD patients are also in a heightened need of a PM approach in the selection of an optimal targeted therapeutic strategy. Based on their phenotype, the three large subcategories of MAFLD can be distinguished as patients with prominent obesity and dysmetabolic conditions (OAFLD) with marked sarcopenia (SAFLD) and with lipodystrophy (LAFLD) [3]. 

Distinguishing between MAFLD subcategories is an important step forward in the PM era since each group has a different prognosis and distinct therapeutic needs. Specifically, in OAFLD patients, weight and metabolic regulation is a pillar in their treatment [3], starting with vigorous lifestyle recommendations. More specifically, weight reduction has major beneficial effects on FLD to ameliorate liver enzymes, decrease steatosis, and histologic findings improvement [46]. In addition, adherence to personalized dietary recommendations, such as the Mediterranean diet, has been inversely associated with steatosis and fibrosis progression, as well as with a decrease in T2DM and cardiovascular risk in these patients [47]. However, rigorous randomized controlled trials are still needed to conclude the effect of dietary patterns (low calories, low carbohydrates, low fat) on FLD progression [48]. In addition, the performance of regular physical activity and increasing muscle mass would benefit FLD management, especially for those with SAFLD [10,47].

After lifestyle recommendations, pharmacological agents such as incretins (GLP-1, GIP, Glucagon alone or most likely in combinations), receptor agonists, sodium–glucose cotransporter-2 inhibitors (SGLT2), PPARγ,α/δ, α/γ, α/δ/γ receptor agonists, DPP-4 inhibitor, statins, or bariatric surgery are possible management options for MAFLD. More specifically, for those with OAFLD with T2DM, the gut-derived hormone glucagon-like peptide-1 (GLP-1) alone or combined with other incretin analogs, such as the glucose-dependent insulinotropic polypeptide, lead to a favorable weight loss, decreased insulin resistance, and a decrease in transaminase levels and liver fat content [14,16,49,50,51]. In the absence of T2DM and in the presence of prediabetes, vitamin E is recommended [50,52]. For subjects with MAFLD and T2DM, without excessive body weight, pioglitazone would be an option that has demonstrated FLD improvement [53].

Recently, clinical trials on FLD have also targeted molecules related to lipid metabolism, which could benefit the FLD and largely the MAFLD population, such as the acetyl-ACC inhibitors, allosteric inhibitors of ACC1 and ACC2, fatty acid synthetase (FAS) inhibitors, SCD inhibitors, AMPK activators, SREBP1-c expression down regulators, LXR and SREBP-1c inhibitors, 11β-hydroxysteroid dehydrogenase type 1 (11β-HSD1/HSD11B1) inhibitors, hydrophilic-non-toxic secondary bile acids, non/steroidal agonist of FXR, thyroid hormone receptor beta (THR-β) agonists, FGF19 analogs, and fibroblast growth factor 21 (FGF-21) receptor agonists. Furthermore, other possible pharmacological targets are under development in preclinical stages, i.e., ALOX12-ACC inhibitors, hepatic stimulator substances (HSS), ACLY inhibitors, SREBP inhibitors, leukemia inhibitory factor (LIF) adipocytes targets, and P53 agonists [51]. The full spectrum of compounds currently in development is covered elsewhere.

It is also known that subjects with LAFLD and low leptin levels may have potential benefits from the pharmacological administration of leptin or leptin analogs. Whether these may decrease the liver fat content and improve many of their metabolic markers in LAFLD remains to be thoroughly studied [3]. Regarding SAFLD, possibly novel pharmacological agents targeting muscle mass, such as the human monoclonal antibody against activin type II receptors, bimagrumab, or similar, are proposed to benefit subjects with SAFLD but need to be further studied in this population [3,54].

The selection of a distinct therapeutic strategy for subjects with FLD is anticipated to significantly improve the safety and efficacy of therapies and, by extension, the quality of life and long-term outcomes in these patients. Finally, it is crucial to consider systemic risk factors associated with MAFLD and the progression of associated comorbidities. Thereby, their follow-up should include routine ASCVD studies, and possibly a multidisciplinary team for controlling the associated cardiometabolic systemic diseases (insulin resistance, dyslipidemia, glucose metabolism alterations, cardiovascular and renal diseases) [2,46].

### 2.3. Advanced FLD (Fibrotic FLD (FAFLD) and End-Stage FLD (ESFLD) in Relation to Precision Medicine

The advanced stages of FLD are the fibrotic FLD (FAFLD) and, subsequently, the end-stage FLD (ESFLD). Fibrosis consists of excessive and unbalanced development of extracellular matrix (EMC) mainly produced from the hepatic stellate cells (HSC) myofibroblast (80–95%) without adequate degradation, leading to its accumulation. Additionally, the EMC composition changes (associated with increased cross-links) and become resistant to degradation during fibrosis [55]. The progression of liver fibrosis involves complex mechanisms associated with multiple pathways (known as multi-hit or multi-parallel hit models), including the metabolic, endocrine, immunological, cellular, and genetic pathways, converging and developing an area of scarring called the fibrotic niche (FN) [56]. Crucial contributors of the FN include distinct macrophage subpopulations, hepatic endothelial cells, mesenchymal cells with PDGFRA/TNFRSF12A expression, and Notch signaling [57]. These contributors act in the context of hepatic oxidative stress developed due to elevated free fatty acids (FFA) and their peroxisomal b-oxidation, with free radicals release that contribute to liver fibrosis development and progression [56]. 

Fibrosis entails pronounced systemic inflammation and parallels the development of portal hypertension and functional hepatocyte insufficiency, as well as the incidence of cardiovascular events, strokes, and metabolic complications. Notably, several studies have shown that fibrosis progression is the principal determinant of increased liver and non-liver-related complications and mortality in NAFLD [55,58]. Therefore, its early recognition is crucial and should start with a clinical evaluation by considering risk factors for the rapid development of fibrosis, such as insulin resistance and T2DM, obesity, sarcopenia, the presence of NASH in non-obese patients, or elevated alanine aminotransferase (ALT) levels above the normal upper limits [59], which should be confirmed with diagnostic tests [60].

#### Currently Available Diagnostic Tests

Although liver biopsy is the gold standard for the diagnosis of NAFLD, non-invasive, safer diagnostic images and scores incorporating biochemical and clinical data are widely available but not as optimal to date [60,61,62]. For instance, ultrasonography (US) is a frequently used non-invasive modality for steatosis screening, but not as sensitive. Less frequently used approaches include the hepatorenal ratio (HRR), which measures the brightness of the renal and liver parenchyma with computer software to estimate the steatosis amount, or the acoustic structure quantification (ASQ), which evaluates the homogeneity of the liver tissue. Besides, the attenuation and backscatter coefficients or the controlled attenuation parameters indirectly calculate the liver fat content [63]. Another tool that predicts steatosis is magnetic resonance imaging derived proton density fat fraction (MRI-PDFF) that predicts steatosis; however, it is less accessible and more expensive [64].

In addition, there are several scores aiming at distinguishing the presence of NAFLD and its stages and histological characteristics i.e., steatosis, fibrosis, and inflammation. Specifically for steatosis, indices such as the Hepatic Steatosis Index (HSI), fatty Liver Index (FLI), NAFLD Liver Fat Score (LFS), or the Lipid Accumulation Product (LAP) are commonly implemented clinical indices and have been proposed to be useful as screening tools [65,66,67]. Similarly, available tests for detecting the presence of NASH are the Index of NASH (ION), the acNASH, and ActiTest, NashTest; and for fibrosis detection i.e., the fibrosis-4 Index (FIB-4), AST to Platelet Ratio Index (APRI), BARD score, and NAFLD fibrosis score (NFS). Other non-specific frequently used indices are the triglyceride to Glucose index (TyG), and the transaminase rations (ALT/AST and AST/ALT) [68,69,70,71,72,73,74,75,76]. The sensitivity and specificity of all these indices are not ideal, and efforts to improve them are underway.

Fibrosis is the most crucial determinant of adverse liver-related and extra-hepatic outcomes in NAFLD [77,78]. As previously mentioned, indices such as FIB-4 are widely used in the clinical setting for fibrosis diagnosis and staging [79]. It has been reported to be a cost-effective and highly sensitive tool to exclude patients with advanced fibrosis [79], and was found to be superior to NFS, APRI, and other scores such as AST to ALT Ratio, Cirrhosis Discriminant Score (CDS), Goteborg University Cirrhosis Index (GUCI), and BARD score, as a predictor of fibrosis [75]. However, FIB-4 and the other indices are also still suboptimal for primary prevention purposes of follow-up, mainly due to the relatively high percentage of false-negative results [80]. Other tools for fibrosis detection include im-aging modalities and biochemical biomarkers, such as the cytokeratin-18 (CK-18) fragment, FibroScan- AST (FAST) score, ELF score, Fibro Meter, Fibro Test, Hepascore, as well as transient elastography, magnetic resonance (MR) elastography (vibration-con-trolled transient elastography (VCTE)), shear-wave elastography (SWE) (including point and 2D SWE), acoustic radiation force impulse imaging (ARFI), or computed tomography for fibrosis assessment [61,62,81,82,83]; however, they also demonstrate a relatively limited diagnostic performance and cannot replace liver biopsy. Therefore, more precise diagnostic tests, including specific multi-test and probably multi-omics biomarkers, are needed to be developed to increase the diagnostic performance of non-invasive tests (NITs) and thus eventually lead to more accurate diagnoses and disease stratification in the future.

This is a currently very active scientific area, with several groups utilizing novel tools and techniques, including machine learning and artificial intelligence, to generate accurate, sensitive, and specific non-invasive diagnostic and prognostic tests for NAFLD. It is envisioned that at a later stage, these tests will be more personalized, and some of them will apply to specific FLD sub-categories [84,85,86,87,88,89,90,91,92], but this is still work in progress, the results of which are expected with great anticipation. 

## 3. Conclusions

### From the Traditional Clinical NAFLD Approach of Everything Applies to Anyone to the Precision Medicine Approach

The introduction of FLD subcategorization in routine clinical practice represents the next critical step forward in the era of NAFLD. Through the regular use of pathophysiology-defined subcategories, the specific FLD diagnosis and the related prognosis will be much more accurate, the distinct clinical needs of each patient will become easier to recognize, and a better-tailored lifestyle along with personalized therapeutic and preventive strategies will be selected. Significant benefits are also anticipated for the families of patients, both in terms of the early identification of individuals at risk as well as reduced disease burden. These advances, as a whole, will enable not only improved patient care, quality of life, and long-term disease outcomes, but also a considerable reduction in healthcare system costs associated with FLD and more options for better-targeted and more effective treatments in the near future.

## Data Availability

Not applicable.
